# Effect of Sodium Hydroxide and Tripolyphosphate on Curcumin Release from Chitosan-Based Macroparticles

**DOI:** 10.3390/ma16175850

**Published:** 2023-08-26

**Authors:** Alessandro Pistone, Annamaria de Gaetano, Elpida Piperopoulos, Chiara Abate

**Affiliations:** Department of Engineering, University of Messina, Contrada Di Dio, I-98166 Messina, Italy; annamaria.dg@hotmail.it (A.d.G.); elpida.piperopoulos@unime.it (E.P.); chiara.abate@unime.it (C.A.)

**Keywords:** chitosan, curcumin, tripolyphophate, controlled-release drug system

## Abstract

This work deals with the synthesis of bare and curcumin (CUR)-loaded chitosan (CS)-based macroparticles by ionic gelation using sodium hydroxide (NaOH) or sodium tripolyphosphate (TPP). The resulting spherical-shaped macroparticles were studied using various characterization techniques, Scanning Electron Microscopy (SEM), Fourier Transform Infrared Spectroscopy (FTIR), Thermogravimetric Analysis (TGA), and Differential Scanning Calorimetry (DSC). The release of CUR from the CS-based particles with respect to time was analyzed, and the encapsulation efficiency and degree of swelling were studied. All formulations showed excellent CUR trapping efficiency, exceeding 90%. In particular, the TPP-crosslinked macrobeads released 34 wt% of the charged CUR within minutes, while the remaining 66 wt% was released slowly. The results indicate that the correct choice of gelling agent and its concentration leads to spherical particles capable of encapsulating CUR and releasing it in a wide range of kinetics so that macrospheres can be used in different applications.

## 1. Introduction

Chitosan (CS), structurally composed of β-1,4-linked 2-amino-2-deoxy-β-D-glucose and N-acetyl-D-glucosamine units, is a derivate of chitin, the second most abundant polysaccharide in nature after cellulose [1,2,3,4]. Among the various biopolymers, CS has attracted much attention for its remarkable biological and physical properties [4] and exhibits a wide variety of environmentally beneficial properties, such as abundant availability, biodegradability, non-toxicity, biocompatibility, recyclability, physiological inertness, cell adhesion, and stability to air and moisture. It also possesses antitumor, mucoadhesive, immunostimulant, antifungal, antimicrobial, antibacterial, antioxidant, hemostatic, and wound healing properties [1,2,3,4], which make it an ideal candidate for pharmaceutical, and industrial applications [1,2,4,5,6,7,8,9]. In this context, CS can be employed as a recyclable green catalyst [5], in supercapacitors and biopolymer batteries, sensors, and water treatment [2,4]. It also finds wide use in cosmetics, agriculture, food technology as food packaging material, and in the biomedical field, particularly in bioimaging, tissue engineering, wound dressing, and the textile industry, as well as in the design and development of drug delivery systems, implants, contact lenses and protein binding [1,2,3,4,10,11,12,13]. In particular, to design controlled release systems of CS-based drugs, the poor barrier and mechanical properties of CS can be modulated and improved by appropriately acting on the crosslinking process, and/or incorporating materials into CS films [3,6].

**Scheme 1:**
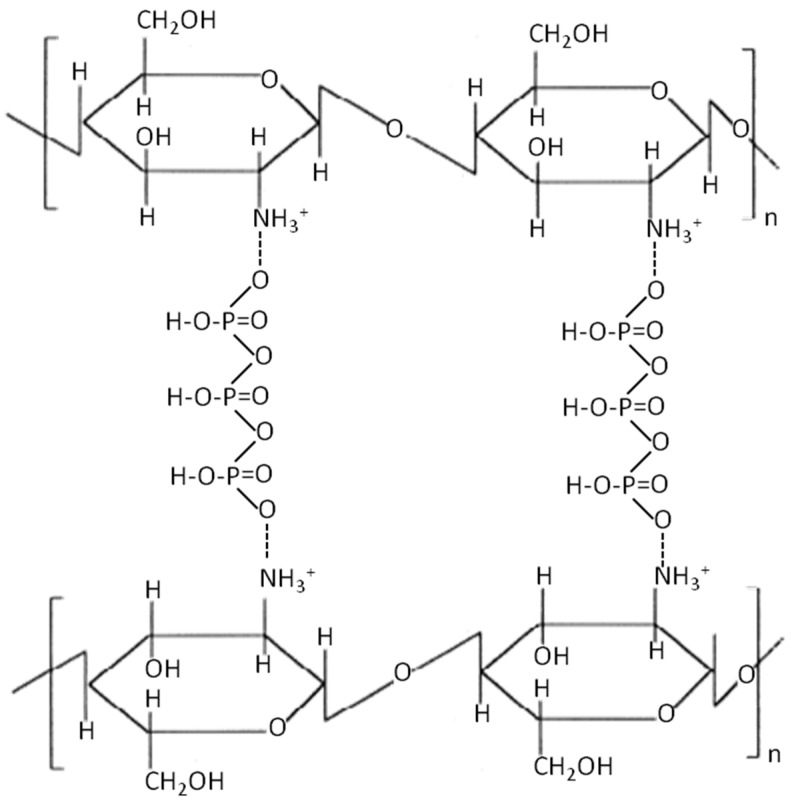
Schematic representation of ionic gelation method.

Once CS is solubilized in aqueous solutions under acid conditions, by protonation of its amine groups, it forms a sol in acetic acid, which can be precipitated in alkali to obtain spherical beads or physical hydrogels; in fact, the basic environment progressively neutralizes the protonated primary amine functions of chitosan promoting the deprotonation of amino groups and therefore a decrease in the polymer ionization and, in turn, of the apparent charge density of chitosan chains. Upon exceeding a critical value, the sol–gel transition occurs, wherein hydrophobic interactions and hydrogen bonds both act to the formation of the three-dimensional network of polymer chains due to physical junctions among entangled chitosan chains [6,8,14]. The use of some cross-linking reagents, such as sodium tripoliphosphate (TPP), is affordable because it involves an easy-to-prepare procedure and without toxic chemicals. In fact, TPP is a nontoxic multivalent anion forming crosslinks by ionic interaction between its negatively charged counter ion and the protonated amine groups of CS, improving the cross-linking density and particle size by adjusting their concentration, and the bioavailability, stability, and controlled release of drugs, thus showing promise as delivery systems for anticancer drugs, proteins, and nucleic acids [6,11,15,16]. Scheme 1 shows a representation of the ionic gelation method.

The practical application of CS-based macro-, micro-, or nano-particles produced by ionic crosslinking is attracting attention both commercially and industrially [17] because of their intriguing properties for environmental applications, such as in catalytic processes [18,19], contaminant removal [13,14], probiotic encapsulation and release under acid conditions [20], and in biomedical field, where the design of CS-carriers is on the rise because CS behaves as an ideal pH-responsive and sensitive carrier for delivering active ingredients due to the presence of positively charged pendant amino groups [10,17,21], and it is also able to encapsulate and release an active principle, such as curcumin.

Curcumin (CUR), an orange-yellow hydrophobic polyphenol derived from the rhizomes of the herb *Curcuma longa*, is another natural bioactive and therapeutic compound due to its anticarcinogenic, antibacterial, antimicrobial, antifungal, antiviral, antimalarial, antioxidant, antimutagenic, cicatrizing, anti-inflammatory, anti-coagulant, anti-fertility, antiprotozal, antifibrotic, antivenom, antiulcer, hypotensive, and anticholesteremic properties, as well as its free-radical effects [3,5,22,23,24]. Because of its flavoring properties, CUR is used as a food coloring, and as a traditional medicine in India and China for the treatment and prevention of several diseases, such as neurological, oncological, autoimmune, metabolic, cardiovascular, and diabetes, due to its extraordinary activities [3,4,5,23], which have attracted increasing interest from researchers to CUR [25]. However, the use of CUR in therapeutic applications has been limited due to its poor bioavailability and water solubility [4,26,27]. In fact, CUR belongs to Class IV of the Biopharmaceutics Classification System (BCS), whose solubility is about 7.8 μg mL^−1^, which can be improved by various strategies of synthesizing appropriate formulations (e.g., solid lipid nanoparticles, solid dispersion, colloidal drug delivery systems, microemulsions, and multi-component crystals) [24]. Encapsulation of CUR within materials, such as CS-based materials, is a strategic approach to improve its dispersibility in aqueous media, chemical stability, controlled release ability, bioactivity, and, thus, use in therapeutic treatment [4,26,28,29]. By way of example, Asif et al. [30] reported the synthesis, characterization, and in vitro study of CUR-loaded CS-nanoparticles to enhance the solubility and bioavailability of CUR and to determine the anti-inflammatory and anti-arthritic activities. As the bioavailability of CUR was found to be enhanced by the nanoparticles, their efficacy was improved and the dosing frequency was reduced [30]. Additionally, Duse et al. [31] studied CUR-loaded CS nanoparticles to enhance the solubility and efficacy of CUR against cancer cells. Therefore, the combination of CUR with a biocompatible matrix, such as CS, enables the production of materials with unique and biologically interesting properties.

This work aimed at developing pH-responsive CS-based beads via a simple, by-product-free dropping method while also studying the effect of the gelling agent on CS-based particles, intending to provide the proper insights for improving the bioavailability and controlled release of the active ingredient, CUR, in CS-based systems, whose actions can in turn be enhanced and intensified by synergy with CUR itself. This work also provides a promising avenue to construct highly biocompatible and biodegradable CS-based macrobeads for use in various practical applications, such as environmental, biomedical, and pharmaceutical, as both CS and CUR are sufficiently safe for clinical drug delivery and tissue engineering applications.

## 2. Materials and Methods

### 2.1. Materials

Chitosan (CS, powder with molecular weight (MW): ~200,000 g mol^−1^ and deacetylated degree ≥ 90%) and sodium tripolyphosphate (TPP, MW: 367.86 g mol^−1^, >95%) were purchased by Glentham LIFE SCIENCES (Corsham, United Kingdom). Sodium hydroxide (Sharlab, Barcelona, Spain), pellets, reagent grade, Scharlau basic (MW: 40 g mol^−1^, ≥97%) were also used. Curcumin (CUR, powder with MW: 368.38 g mol^−1^) was obtained from labfor. Ethanol (EtOH, MW: 46.07 g mol^−1^, ≥97%) and methanol (MW: 32.04 g mol^−1^, ≥99.9%) were purchased from Honeywell Research Chemicals (Seelze, Germany); acetic acid glacial (MW: 60.05 g mol^−1^, ≥99.8%) and acetonitrile (MW: 41.053 g mol^−1^, ≥99.9%) from Carlo Erba reagents (Cornaredo, Italy). Hydrochloric acid (HCl, 37%) was also purchased from Sigma-Aldrich ACS reagent (Milan, Italy), and phosphate-buffered saline (PBS) from Sigma-Aldrich (Milan, Italy). Double-deionized water (conductivity: <0.1 μS cm^−1^) was employed. All reagents were used without further purification.

### 2.2. Preparation of the CS- and CS- CUR- Based Macrobeads

Three different gelling media were used to prepare the unloaded and CUR-loaded CS-based macroparticles: (a) 4 wt% NaOH in EtOH (26% *v*/*v*) solution (250 mL, pH~12); (b) 2 wt% TPP; and (c) 5 wt% TPP in aqueous solutions (250 mL, pH~8).

In particular, 0.5 g of chitosan (CS) was gradually dissolved in 50 mL of an acetic acid solution (2%, *v*/*v*) under magnetic stirring for 3 h at room temperature (rt). Then, the CS solution was dropped with a peristaltic pump (0.1 mL min^−1^), using a needle from a syringe with an internal diameter of 0.4 mm) into solutions (a), (b), and (c) under magnetic stirring (550 rpm). The obtained samples were denoted as CS-Na, CS-TPP2, and CS-TPP5, respectively, and shown in Table 1.

**Figure 1:**
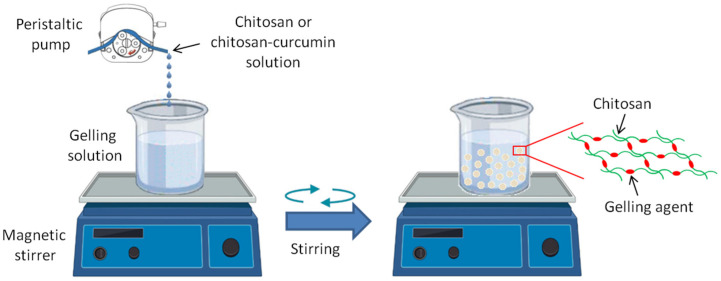
The illustrative procedure of preparing chitosan (CS)-based particles by the ionic gelation method using NaOH or TPP.

To prepare CS-based macroparticles with CUR, 0.5 g CS was dissolved in 50 mL of an acetic acid solution (2%, *v*/*v*) under magnetic stirring for 3 h (rt). Thereafter, 0.1 g of CUR was completely dissolved in 25 mL of a mixture of acetonitrile/methanol (1:1 *v*/*v*), filtered, and stored in the dark. Then, the CUR mixture was dropped into the CS solution and kept under magnetic stirring (550 rpm) for 30 min. The preparation procedure of the CUR-loaded CS-based macrospheres is similar to that of the unloaded samples described previously and shown in Figure 1. In more detail, the CS-based particles, loaded with CUR, prepared by precipitation in (a), (b), and (c) solutions, are CS-Na-CUR, CS-TPP2-CUR, and CS-TPP5-CUR, respectively. All sample codes and their compositions are given in Table 1. After the cross-linking procedure, the macrobeads were sonicated for 1 h (rt), filtered, and washed with double-distilled water to neutral pH. Finally, they were stored at 4 °C.

### 2.3. Characterization of the CS- and CS-CUR Macrobeads

The prepared macroparticles were observed with an ECLIPSE *Si* Upright light Microscope (Nikon, type 104c, Amstelveen, The Netherlands) and analyzed by Scanning Electron Microscopy (SEM; FEI Quanta 450 equipment, Hillsboro, OR, USA). The samples were completely dried (rt) and covered with a carbon coating to ensure good conductivity of the electron beam; then, photographs were taken with an accelerating voltage of 5 kV. The average diameter of the macroparticles was estimated by collecting more than 200 images in different areas of the samples.

FTIR spectra were obtained with a Perkin Elmer spectrometer (Perkin Elmer, Waltham, MA, USA), Spectrum Two model. Spectra were previously collected by mixing a small amount of macroparticles with KBr and compressing them to form tablets. IR spectra were obtained in absorbance mode in the spectral region 4000–450 cm^−1^ with a resolution of 4 cm^−1^.

Thermogravimetric studies were performed from 150 to 800 °C at 10 °C min^−1^ under argon on a TAQ500 instrument (TA Instruments, New Castle, DE, USA).

An SDT-Q 600 calorimeter (TA Instruments, New Castle, DE, USA) was used for DSC characterization. DSC curves were obtained using aluminum crucibles containing about 5 mg of sample under a nitrogen atmosphere (flow rate: 50 mL min^−1^) from 50 to 400 °C at 10 °C min^−1^. An empty aluminum crucible was used as a reference. The DSC cell was calibrated with indium (melting point 156.6 °C; ΔH_fusion_ = 28.54 J g^−1^) and zinc (melting point 419.6 °C).

CUR encapsulation efficiency was measured by the total dissolution of 50 g of macroparticles in 100 mL of HCl solution (0.1 mol L^−1^). After filtration, the concentration of encapsulated CUR was determined on a Shimadzu UV-Visible spectrophotometer (model UV-2401 PC, Shimadzu, Milan, Italy), set at 429 nm. A standard sample of CUR dissolved in a solution of acetonitrile/methanol (1:1 *v*/*v*) was used to obtain the following calibration equation, obtained in the CUR range between 0.00625 g L^−1^ and 0.05 g L^−1^:

y = 4.8865x + 0.0273(1)

where R^2^ = 0.9942, x is the CUR concentration (g L^−1^), and y is the absorbance at 429 nm. Encapsulation efficiency was expressed as the percentage of encapsulated CUR relative to its total amount loaded into the CUR solution before the gelling step. Loading efficiency tests were assayed in triplicate for each sample.

CUR release kinetics were studied by suspending 50 g of macroparticles in 100 mL of PBS at pH 7.4. At predetermined time intervals, 3 mL of sample was withdrawn to determine the quantity of released dye and an equivalent amount of fresh dissolution medium was used to replace that removed. The samples were analyzed by UV-Vis spectrophotometry, as previously discussed. All experiments were performed in triplicate and the results were expressed as cumulative CUR release.

The swelling properties of the macroparticles were studied as gravimetrically measured water uptake by treating the macroparticles in PBS under gentle stirring for 120 min. The swollen samples were removed periodically (0, 5, 15, 30, 45, 60, 90, and 120 min), and their net weight was determined by weighing them after removing the adsorbed water on the surface by blotting them with filter paper. Each swelling experiment was repeated twice, and the average value was taken as the swelling degree, calculated by the following formula:

Swelling degree = [(M_t_ − M_0_)/M_t_] 100 (2)

where M_t_ is the weight of the swollen sample at time t, and M_0_ is the initial weight of the sample before immersion in double-distilled water.

## 3. Results

### 3.1. Morphological Characterization of the Macrobeads

**Figure 2:**
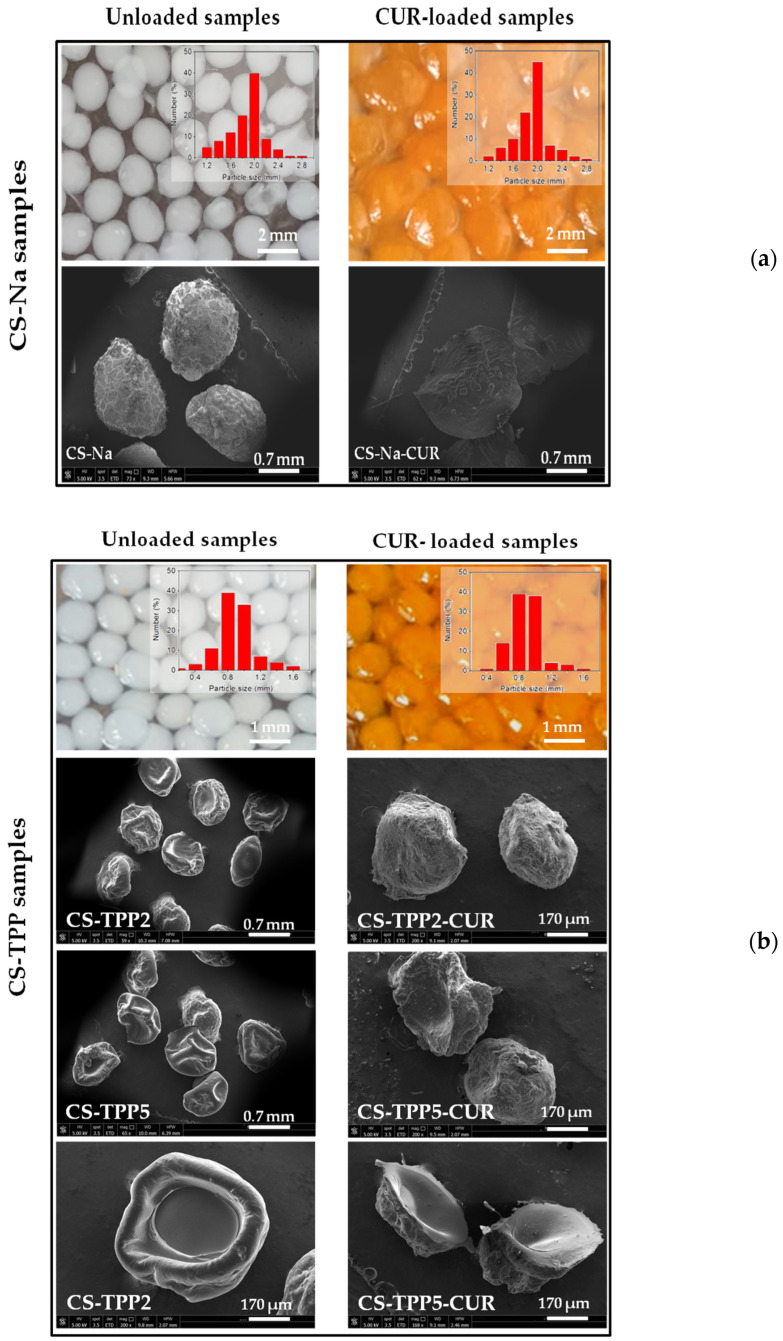

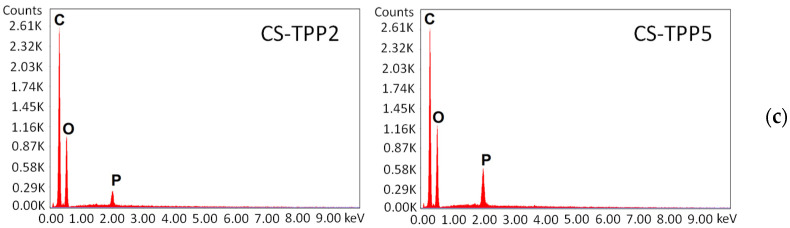
Optical images and SEM micrographs of CS-based macroparticles obtained in NaOH (**a**) CS-Na and CS-Na-CUR, and crosslinked with TPP (**b**) CS-TPP2, CS-TPP2-CUR, CS-TPP5, and CS-TPP5-CUR, and (**c**) EDX analyses of CS-based macroparticles crosslinked with TPP.

The morphological properties of all CS-based macroparticles were investigated. Figure 2 shows optical images and SEM micrographs of CS-based macroparticles obtained in NaOH, (a) CS-Na, and CS-Na-CUR, as well as those with TPP (b), CS-TPP2, and CS-TPP2-CUR, compared with SEM micrographs of CS-TPP5 and CS-TPP5-CUR. All wet particles had a spherical shape and uniform size distribution. In particular, CS-Na and CS-TPP2 were white in color and had a better spherical distribution than the CUR-loaded macroparticles, which were yellow-brown. The optical images of CS-Na and CS-Na-CUR showed an average diameter of 1.9 mm, which decreased to 0.9 mm in those of CS-TPP2 and CS-TPP2-CUR. SEM analysis showed that all particles had a rough outer surface, and an increase in roughness was observed in CS-CUR particles, particularly CS-TPP2. The morphological differences in terms of increased roughness observed by the SEM analyses can be attributed to greater difficulty in the structural rearrangement of the biopolymer chains due to the presence of additives or to increased crosslinker load, as observed by Martins et al. [32]. The macrobeads crosslinked with TPP also exhibited cavities with a very smooth inner surface. Figure 2c also describes EDX analyses of CS-based macroparticles crosslinked with TPP, showing different intensities of phosphorus signals between the CS-TPP2-CUR and CS-TPP5-CUR samples and confirming the increased TPP burden in CS-TPP5-CUR compared to CS-TPP2-CUR, as expected.

### 3.2. FTIR Analyses

**Figure 3:**
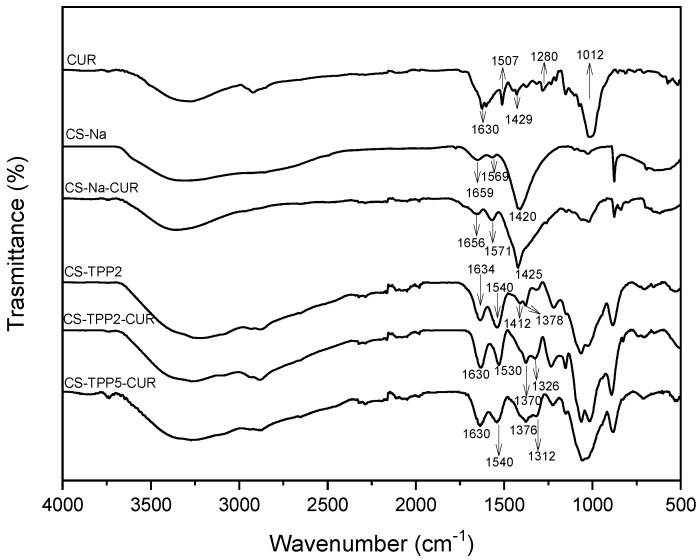
FTIR spectra of the investigated samples.

The structure of the CUR, CS, and CS-CUR macroparticles, obtained in the different gelling media, was also confirmed by FTIR spectroscopy. Figure 3 shows the resulting FTIR spectra. The FTIR spectrum of CUR shows a characteristic broadband in the region between 3500 and 3200 cm^−1^, due to the phenolic O-H stretching vibration, and the absorption band at 2922 cm^−1^, referring to the stretching vibration mode of the C-H bond [33]. As previously reported in the literature [25,34], the peaks at 1630 cm^−1^ and 1600 cm^−1^ are assigned to the aromatic moiety C=C stretching and the benzene ring stretching vibrations, respectively. At 1507 cm^−1^ the C=O and C=C vibrations occurred, while the olefin C-H bending vibrations were at 1429 cm^−1^. The peaks at 1280 cm^−1^ and 1012 cm^−1^ are due to the asymmetrical stretching vibration of C-O-C and the symmetrical C-O-C stretching vibration of aryl alkyl ether, respectively [33,34]. The sharpest peaks located in the range of 700–900 cm^−1^ are attributable to the C-H deformation of the alkene group [33].

The FTIR spectra for CS macroparticles were different in the two gelling media investigated. In fact, the CS-Na spectrum showed characteristic absorption bands from 3600 to 3000 and at 2867 cm^−1^, related to the presence of the OH and CH_3_ groups, respectively [35]. The peaks at 1659 cm^−1^ and 1569 cm^−1^ are attributable to the C=O stretching in the structure of N-acetylglucosamine and NH_2_ stretching of glucosamine, respectively; the peak at 1420 cm^−1^ is due to the symmetrical carboxylate anion stretching [36]. The CS-TPP2 spectrum is very similar to that of the CS-TPP5 macroparticles, which is neglected in Figure 3 for simplicity. The FTIR spectrum of CS-TPP2 showed an absorption band centered at 3219 cm^−1^ and another one at 2874 cm^−1^, and the two peaks at 1634 cm^−1^ and 1540 cm^−1^ attributable to the C=O stretching in the structure of N-acetylglucosamine and NH_2_ stretching of glucosamine. In addition, the peaks at 1412 cm^−1^ and 1378 cm^−1^ occurring in the FTIR spectrum of CS-TPP2 are due to the interaction between phosphate groups and protonated CS [37,38], while peaks at 1218 cm^−1^ and 1150 cm^−1^ represent stretching vibrations, ascribable to the phosphate groups linked to the CS through intermolecular interactions [35,39]. Moreover, in all FTIR spectra, there was a broad absorption band in the 3450–3200 cm^−1^ region, which can be attributed to the overlapping stretching vibrations of the hydroxyl group and the amino group [35]. In CS-based macrobeads loaded with CUR, a blue shift was found that could be attributed to hydrogen bonds between the CUR and CS molecules, both in the FTIR spectrum of CS-Na-CUR and in that of CS-TPP2-CUR and CS-TPP5-CUR. The interaction was also confirmed by the increased N-H bond length and blue shift in CS-based samples loaded with CUR compared with the spectrum of the uncharged ones [35]. These effects were more pronounced in CS-based macrobeads cross-linked with TPP.

### 3.3. TGA and DSC Analyses

**Figure 4:**
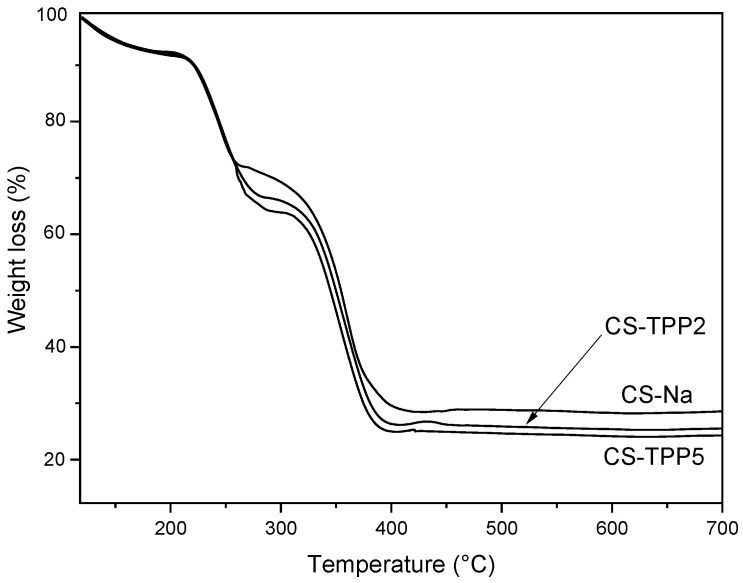
TGA analyses of the CS-based macrobeads.

The thermal stability of CS-based macroparticles was studied by TGA analysis, under settings reported in Section 2.3. Figure 4 shows the profiles of CS-based macrobeads that, regardless of the type of gelling agent, have similar thermal degradation behavior. Two main steps of weight loss (%) were observed for all samples; the first, located at about 240 °C, is assigned to the dehydration of saccharide rings, and the second stage, at about 350 °C, is related to the pyrolytic decomposition of the polymeric units with the formation of the charcoal residues. In particular, for CS-Na, the weight loss was ~22% at about 240 °C, and ~29% at about 350 °C. For CS-TPP2 and CS-TPP5, the former weight loss was ~27% and ~30%, and the latter was 40%. Increasing the amount of TPP in the samples, a slight decrease in the thermal stability (~3%) of the samples was observed. In fact, gelling with TPP led to a lowering of the thermal stability compared to the macrobeads obtained with NaOH. Similar profiles were reported by Laus et al. [38] and were attributed to a reduction in the crystallinity of the polymeric network formed by the cross-linked CS chains with bulky phosphate groups.

**Figure 5:**
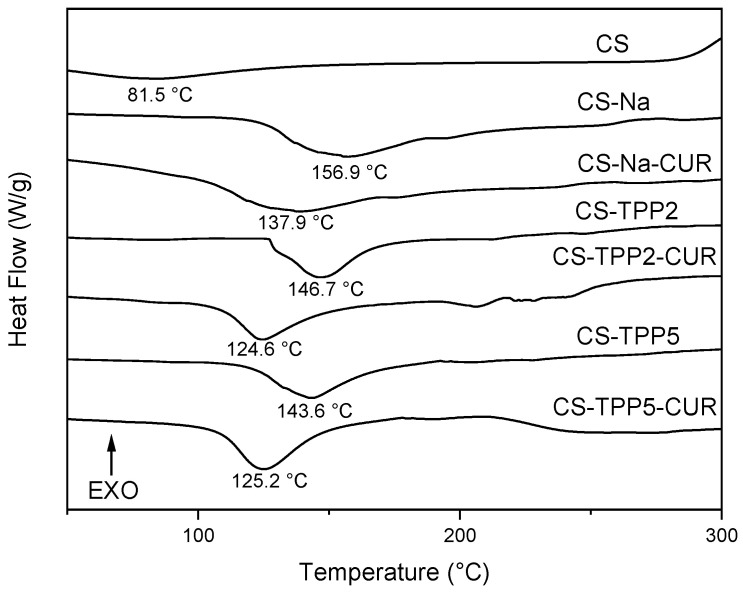
DSC curves of all investigated samples.

DSC was employed to study the thermal effect of physical and chemical changes; Figure 5 reports the DSC curves of all the samples investigated. Pure CS showed heat absorption over a wide temperature range centered at 81.5 °C due to the removal of physisorbed water. CS-based macrobeads obtained in the two gelling media exhibited an important endothermic signal at about 150 °C due to the glass transition temperature (T_g_) of the CS-based biopolymer. In particular, the CS-based macrobeads crosslinked in TPP media display a slightly lower T_g_ than those prepared using NaOH, whose T_g_ value was 156.9 °C and dropped to 146.7 °C in CS-TPP2 and 143.6 °C in CS-TPP5, respectively, indicating easier movement of the TPP-cross-linked CS chains. The addition of CUR led to a further lowering of T_g_, from 137.9 °C in CS-Na-CUR to 124.6 °C in CS-TPP2-CUR and 125.2 °C in CS-TPP5-CUR. This effect shows that CUR is trapped between the CS chains, thus acting as an additional spacer of the CS chains and facilitating their further mobility [40].

### 3.4. Evaluation of Encapsulation and Swelling Degree of CUR

The encapsulation efficiency of CUR in the various CS-based macrobeads was measured as reported in Section 2.3, and the resulting data are listed in Table 2.

The results indicated that the loading efficiency was very high for all the samples, ranging from about 100 wt% using NaOH to 92–96 wt% with TPP as a gelling agent. A slight decrease in encapsulation efficiency was observed with increasing TPP concentration in the gelling solution, from 95.9 wt% to 91.4 wt% for CS-TPP2-CUR and CS-TPP5-CUR, respectively. This decrease may be due to the amount of CUR trapped in the network of CS that forms the particle walls rather than CUR remaining encapsulated in the inner cavity of the particles. An increase in the TPP concentration leads to a more cross-linked network within the particle walls, with more phosphate groups involved, which in turn hinders CUR encapsulation due to steric hindrances. This reduction in drug encapsulation efficiency in the CS/TPP system has already been observed [41,42,43,44].

**Figure 6:**
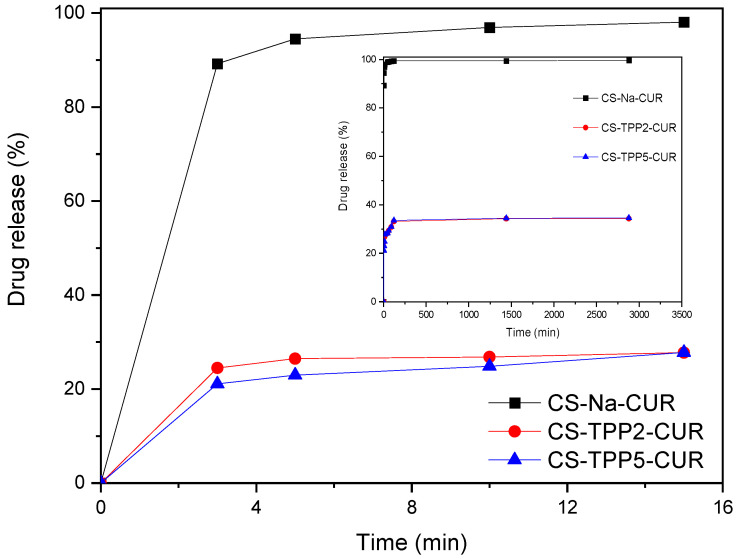
CUR release profiles of the macrobeads obtained with different gelling agents.

Figure 6 shows the effect of the different gelling agents on CUR release behavior. All samples analyzed showed a rapid release profile within minutes, but large differences were observed between particles obtained with NaOH or TPP as a gelling agent. In particular, the CS-Na-CUR sample released 95 wt% of the loaded CUR within 5 min, reaching full release of the charged CUR at 120 min. The CS-based crosslinked with TPP showed a rapid release of about 27 wt% and 23 wt% of the loaded CUR within 5 min for CS-TPP2-CUR and CS-TPP5-CUR, respectively, followed by a slower release of no more than 34 wt% of loaded CUR after 2 days. This similar profile is depicted in Figure 6 (red and blue curves of the inner part). Thus, the remaining amount of loaded CUR (66 wt%), strongly trapped in the CS chains or the inner cavity of the particles, will be released with very slow kinetics. The rapid release observed in CS-Na-CUR-based systems, unlike that observed in TPP-crosslinked systems, is probably attributable to the gelling process carried out without the use of a crosslinking molecule, as is the case with TPP-based systems. Other authors have observed that increasing the concentration of the gelling agent leads to a decrease in the drug released due to the reduction of drug diffusion from the CS network [45].

Table 2 also shows the swelling degree of the samples obtained with different gelling agents, which was not detectable for CS-Na-CUR. Indeed, it showed rapid CUR release and subsequent degradation effect in PBS, and, therefore, it was difficult to fix its degree of swelling at equilibrium. For the samples obtained with TPP, the swelling ability decreased with increasing TPP concentration, suggesting that a more tightly cross-linked CS matrix does not swell as much as a poorly cross-linked CS matrix. The swelling behavior also reflects that of drug release, as also reported by Katas et al. [46].

The higher swelling degree of CS-TPP2-CUR (120 wt%), compared to that of CS-TPP5-CUR (90 wt%), could be due to the greater penetration of medium in the polymer matrix, resulting in a better drug release from the particle structure (Figure 6).

## 4. Conclusions

In the present work, spherical chitosan (CS)-based particles were synthesized by the ionic gelation method using two different gelling agents, NaOH and tripolyphosphate (TPP). The latter was employed at 2 wt% and 5 wt%, while NaOH at 4 wt%. Under the same conditions, the CS-based particles were loaded with curcumin (CUR). The obtaining macrobeads, namely CS-Na, CS-TPP2, CS-TPP5, CS-Na-CUR, CS-TPP2-CUR, and CS-TPP5-CUR, were characterized by SEM, FTIR, and TGA and DSC analyses. SEM confirmed the spherical morphology of the macroparticles, with a rough outer surface and a smooth inner cavity, while the increased TPP-loading in CS-TPP5-CUR was verified by EDX analyses.

The FTIR spectra showed that the interaction between CS and CUR occurred, presumably through hydrogen bonding, while the TGA and DSC analyses confirmed the encapsulation of CUR in the CS-based particles. In particular, for all investigated samples, the encapsulation efficiency of CUR was higher than 92 wt% and CUR serves as an additional spacer of the CS-chains and makes their movement and mobility easier, especially in the TPP-cross-linked CS-based macroparticles.

There have been several releases of CUR from the samples tested. On one hand, CS-Na-CUR allows for the very rapid release of CUR probably due to the gelling process carried out without the use of a cross-linking molecule, as is the case with TPP-based systems instead; on the other hand, the TPP-cross-linked particles released 34 wt% of the charged CUR within minutes. This effect is also in accordance with the swelling degree (wt%), determined in the TPP-cross-linked CS-based macroparticles. Notably, the swelling degree of CS-TPP2-CUR and CS-TPP5-CUR was found to be 120 wt% and 90 wt%, respectively, demonstrating that it decreases as the TPP concentration increases. This trend could be due to the increased penetration of the medium into the polymeric network of CS-TPP2-CUR than CS-TPP5-CUR, resulting in their higher CUR drug release from the particle structures.

The results indicated that the correct choice of gelling agent and its concentration leads to the formation of spherical CS-based macroparticles capable of encapsulating CUR and releasing it in a broad spectrum of kinetics. However, in this paper, the use of harmful solvents, such as the acetonitrile/methanol mixture used to dissolve CUR, limits the application of CS-based macrospheres in clinical settings. Therefore, further studies specifically targeting the comparative use of harmless solvents, to improve the bioavailability and solubility of CUR under physiological conditions, are needed for the subsequent evaluation and determination of the potential of CS-based macroparticles as attractive drug delivery systems.

## Figures and Tables

**Table 1:** Sample codes and composition of chitosan (CS)-based macroparticles.

Sample Code	CS (g)	Gelling Agent	CUR (g)
CS-Na	0.5	NaOH 4 wt%	-
CS-Na-CUR	0.5	NaOH 4 wt%	0.1
CS-TPP2	0.5	TPP 2 wt%	-
CS-TPP2-CUR	0.5	TPP 2 wt%	0.1
CS-TPP5	0.5	TPP 5 wt%	-
CS-TPP5-CUR	0.5	TPP 5 wt%	0.1

**Table 2:** Encapsulation efficiency and swelling degree of CUR in the CS-based macroparticles.

Sample Code	Encapsulation Efficiency (wt%)	Swelling Degree (wt%)
CS-Na-CUR	99.8	n.d.
CS-TPP2-CUR	95.9	120
CS-TPP5-CUR	91.4	90

n.d.: not detectable.

## Data Availability

The data presented in this study are available on request from the corresponding author.
